# Bootstrapping complex time‐to‐event data without individual patient data, with a view toward time‐dependent exposures

**DOI:** 10.1002/sim.8177

**Published:** 2019-06-04

**Authors:** Tobias Bluhmki, Hein Putter, Arthur Allignol, Jan Beyersmann, on behalf of the COMBACTE‐MAGNET consortium

**Affiliations:** ^1^ Institute of Statistics Ulm University Ulm Germany; ^2^ Department of Medical Statistics and Bioinformatics Leiden University Medical Center Leiden The Netherlands; ^3^ Merck KGaA Darmstadt Germany

**Keywords:** bootstrap, internal time‐dependent covariates, joint model, simulation, survival analysis

## Abstract

We consider nonparametric and semiparametric resampling of multistate event histories by simulating multistate trajectories from an empirical multivariate hazard measure. One advantage of our approach is that it does not necessarily require individual patient data, but may be based on published information. This is also attractive for both study planning and simulating realistic real‐world event history data in general. The concept extends to left‐truncation and right‐censoring mechanisms, nondegenerate initial distributions, and nonproportional as well as non‐Markov settings. A special focus is on its connection to simulating survival data with time‐dependent covariates. For the case of qualitative time‐dependent exposures, we demonstrate that our proposal gives a more natural interpretation of how such data evolve over the course of time than many of the competing approaches. The multistate perspective avoids any latent failure time structure and sampling spaces impossible in real life, whereas its parsimony follows the principle of Occam's razor. We also suggest empirical simulation as a novel bootstrap procedure to assess estimation uncertainty in the absence of individual patient data. This is not possible for established procedures such as Efron's bootstrap. A simulation study investigating the effect of liver functionality on survival in patients with liver cirrhosis serves as a proof of concept. Example code is provided.

## 1 INTRODUCTION

Simulation studies have become a crucial tool to systematically compare the performance and properties of statistical methods in relation to the truth. The ideal scenario generates biologically plausible data following a motivating real‐world situation.^1^ Efficient and easy‐to‐implement simulation algorithms to generate survival data in the presence of a set of time‐independent covariates are well established. Standard toolboxes allow for a variety of parametric choices for the baseline hazard function while incorporating covariate effects via the semiparametric framework of the Cox proportional hazards model or extensions thereof.^2^, ^3^, ^4^, ^5^ Since, nowadays, time‐to‐event outcomes with longitudinal covariate patterns are frequently encountered in biomedical studies, there has been considerable research on flexible data‐generating procedures. For instance, Leemis et al studied simulation for the Cox model with time‐varying covariates and generated the survival time by inverting the cumulative survival hazard given the covariate trajectory.^6^, ^7^, ^8^ Various refinements have been suggested by Austin^9^; see also the work of Rivera and Lumley for a recent application.^10^ In more complex settings where the cumulative survival hazard function is noninvertible or analytically intractable, root finding with nested numerical integration has been suggested.^4^ Other algorithms rely on transformations according to piecewise exponential distributions using rejection sampling.^11^, ^12^, ^13^

However, all these methods have in common that the covariate trajectory is sampled a priori and that the event time is drawn from a *conditional* survival distribution given the future covariate trajectory irrespective of the survival status. One objective of this paper is to suggest a more plausible algorithmic point of view for internal (endogenous) time‐dependent covariates.^14^ The challenge lies in (i) imposing impossible sampling spaces in real life accompanied by (ii) a latent failure time structure with unclear interpretation. Consequently, the hazard specifications used for simulation do not have an interpretation as a population‐level summary. This could, eg, be a concern in the context of study planning, which should be connected to the corresponding statistical analysis. In‐depth discussions regarding the plausibility and identifiability of latent times can be found elsewhere.^15^, ^16^, ^17^, ^18^ A similar argumentation applies to the algorithm proposed by Sylvestre and Abrahamowicz,^19^ which generates survival times and covariates independently and matches them in retrospect according to the permutation probability law corresponding to the partial likelihood of the Cox model.

Instead, we follow the work of Cortese and Andersen and represent each possible value of a time‐dependent covariate as additional (intermediate) transient states in a multistate model.^20^ This procedure has also been mentioned by Andersen^21^ as well as Beyersmann and Schumacher.^22^ Multistate event histories model complex time‐to‐event data as a sequence of transitions (the events) between different states (the event types). Applications in medical research include oncology,^23^, ^24^, ^25^ cardiology,^26^ gastroenterology,^27^ orthopaedics,^28^ psychology,^29^ prenatal studies,^30^ or hospital epidemiology.^31^, ^32^ This paper considers simulation—or resampling—of multistate trajectories from an empirical multivariate hazard measure as, eg, given by the (nonparametric) multistate Nelson‐Aalen estimator of cumulative transition hazards.^33^ The procedure proposed here is the empirical analogue of a probabilistic construction of Gill and Johansen,^34^ who investigated how to express the distribution of a multistate model based on a (parametric) specification of the transition hazards (see also elsewhere for a textbook account^33^). The key is that multistate data can be realized as a nested sequence of competing risks experiments by iteratively generating the waiting *time* in the current state (step 1) and, as an intrinsic part of the model, the event *type* (step 2). Specifically, this concept provides a comprehensible building plan of how the involved hazards interplay in order to obtain multistate trajectories over the course of time. The appeal of this perspective is that it avoids both concerns on identifiability, plausibility, and usefulness known from the latent failure time approach as well as sampling spaces impossible in real life. We also mention an alternative multistate algorithm suggested by Crowther and Lambert^35^, ^36^ and recently applied elsewhere.^37^ Their method allows practitioners to flexibly specify a multivariate hazard measure based on prespecified “marginal” distributions; however, the decision on the transition type within their implementation is again based on a latent time framework. Thus, we will emphasize simulation as well as interpretation along the lines of the work of Gill and Johansen,^34^ because, on the one hand, all generated quantities turn out to have a “natural” (or “realistic”) interpretation in the sense that they are not hypothetical and can be interpreted on the population level. The latter may also be a concern with respect to the current discussion on estimands and post‐randomization events.^38^, ^39^, ^40^, ^41^ On the other hand, it guarantees the natural order of the events, whereas its parsimony follows the principles of Occam's razor^42^ without losing any flexibility.

The advantages of an empirical perspective turn out to be diverse. First, it allows for mimicking complex real‐world time‐to‐event data, avoiding any preprocessing procedures. The latter may be difficult or computationally expensive in practice regarding the derivation of closed forms for all time‐dependent transition hazards. Second, the algorithm works in a time‐discrete setting, which is a typical situation in longitudinal studies resembling the viewpoint in marginal structural models with time‐dependent confounding.^43^ Third, it provides for a convenient resampling (bootstrap) technique not necessarily requiring individual patient data but only the estimated cumulative transition hazards. The latter may be derived from, eg, published Nelson‐Aalen plots. As a consequence, we also suggest the simulation algorithm as a novel bootstrap procedure in order to assess estimation uncertainty as well as a general tool for sample size calculations at the study planning stage (see elsewhere for a recent application in the context of competing risks^44^). Fourth, the algorithm is not only a computational instrument for data generation but also can be used as an operational tool for interpretation by providing additional insight into the data.^45^ Finally, our proposal complements the rich literature on simulating time‐to‐event data in the presence of time‐dependent covariates by avoiding conceptual obstacles when such covariates are internal. Empirical simulation has been investigated in depth and recently applied for the special case of competing risks.^45^, ^46^ For the general multistate framework, it has been briefly suggested in the context of prediction in reduced‐rank Cox models,^47^ but a more comprehensive treatment has not been given.

The remainder of this paper is structured as follows. We start by reviewing the multistate framework in Section 2. Section 3 recapitulates simulation of survival data in the presence of time‐varying covariates on the basis of an illustrative example from oncology.^48^, ^49^ Here, we highlight issues regarding the previously mentioned standard procedures and explain why multistate methodology is an appropriate alternative. Section 4 outlines “empirical simulation.” Section 5.1 provides details about the study example used for the proof of concept given in Section 5.2. It relates to a published randomized clinical trial in liver cirrhosis patients. The primary objective was to show a prolonging effect on the survival of a new hormone therapy. A relevant factor in this context is the prothrombin index measuring liver functionality. To account for its internal time‐dependent nature, we apply an illness‐death multistate model with recovery dichotomizing the index into the categories “normal” and “abnormal.”^50^ Section 5.3 suggests empirical simulation as a novel bootstrap procedure in order to assess uncertainty in parameter estimation. One application may be confidence interval construction in the absence of individual patient data, which is not possible for established procedures such as Efron's bootstrap. A conclusion is in Section 6. Mathematical proofs are deferred to the Appendix. Example code is provided as web‐based Supporting Information.

## 2 THE MULTISTATE FRAMEWORK

Let (*X*_*t*_)_*t* ≥ 0_ be a multistate process with finite state space S and fulfilling the time‐inhomogeneous Markov assumption. The hazards of an *l*→*m* transition (also called transition intensities) *α*_*lm*_(*t*) are nonnegative functions defined as

(1) $$\alpha_{lm}(t)\cdot dt=P\left(X_{t+dt}=m|X_{t-}=l\right),\;l,m\in S,\;l\neq m$$

with cumulative counterparts

(2) $$A_{lm}(t)=\overset{t}{\underset{0}{\int }}\alpha_{lm}(u)du.$$

**Figure 1:**
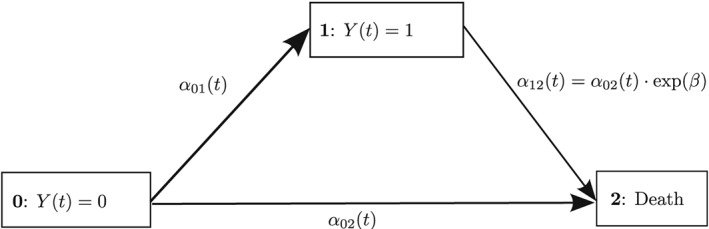
A joint model to assess the effect of a time‐dependent covariate Y(t) ∈ {0,1} on survival: illness‐death multistate model without recovery treating the two covariate levels as separate transient states. Transition hazards α_01_(t),α_02_(t), and α_12_(t) are included

Here, *t*− denotes the time just prior to *t* and *dt* an infinitesimal small time interval. The transition intensity in (1) can be interpreted as the “instantaneous force” to switch from state *l* to state *m* in [*t* + *dt*). State l∈S is called “absorbing” if no transitions out of state *l* are modeled and $\alpha_{lm}(t)\equiv 0,\;\forall t,\;\forall m\in S,\;m\neq l$, and “transient” otherwise. A graphical visualization of a specific multistate model called “illness‐death model without recovery” considered later on is given in Figure 1. The Markov assumption guarantees that the probability of a future transition only depends on the current state *l* and time *t*, but not on the entire history of the process *X* up to time *t*. We will later discuss how to relax the Markov assumption.

The analysis of our motivating study example in Section 5 will also consider transition probabilities. Let $A(t)={\left(A_{lm}(t)\right)}_{l,m\in S}$ be the transition intensity matrix. Its diagonal entries are chosen such that the sum of each row is equal to zero. Then, the matrix of transition probabilities is defined by

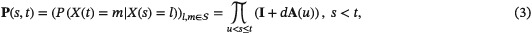

where 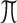 is the product integral and **I** is the identity matrix.^34^ The fundamental nonparametric estimator of the transition probabilities is the Aalen‐Johansen estimator^51^

(4) $$\hat{P}(s,t)=\underset{s<u\leq t}{\prod }\left(I+\Delta \hat{A}(u)\right),$$

where ∏ is a finite product over all observed transition times of any type *u* and $\Delta \hat{A}$ is the matrix of increments of the Nelson‐Aalen estimators of the cumulative hazards (2) with nondiagonal entries

(5) $$\Delta {\hat{A}}_{lm}(t)=\frac{\#\{\text{observed}\;l\to m\;\text{transitions at}\;u\}}{\#\{\text{individuals observed in state}\;l\;\text{just prior to}\;u\}}$$

and diagonal entries $\Delta {\hat{A}}_{ll}(t)=-\sum_{l\neq m}\Delta {\hat{A}}_{lm}(t)$. The Aalen‐Johansen estimator allows the data to be subject to independent left‐truncation (delayed study entry) and right‐censoring mechanisms (see, eg, the book of Aalen et al for details^51^). Note that the increments of the Nelson‐Aalen estimator given relation (5) will play a key role within the nonparametric variant of the simulation algorithm proposed in Section 4. The R packages mstate 52, 53 or etm 54 and mvna 55 may be used for fast computation of $\hat{P}$ and $\hat{A}$.

The Markov assumption can be relaxed, allowing *α*_*lm*_(*t*) to depend on, for instance, the duration time in state *l* via a Cox proportional hazards model, eg, including the state arrival times or the number of previous visits to a state in the linear predictor. The Aalen‐Johansen estimator of the unconditional state occupation probabilities is still a consistent estimator^56^, ^57^ in such non‐Markovian situations at least under random right‐censoring, whereas the estimation of the state occupation probabilities appears to be less sensitive to the Markov assumption than originally thought.^58^, ^59^ An Aalen‐Johansen landmark estimator of **P**(*s*,*t*) for randomly right‐censored non‐Markov models has recently been suggested.^60^

## 3 SURVIVAL HAZARD IN THE PRESENCE OF A TIME‐DEPENDENT EXPOSURE

**Figure 2:**
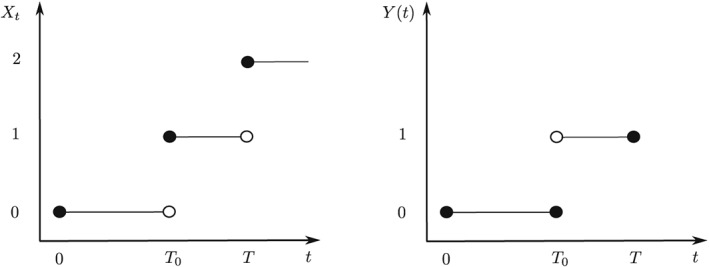
Relation between the multistate process X_t_ and covariate process Y(t). Both T_0_ and T are random variables. X is right‐continuous, but Y is left‐continuous

**Figure 3:**
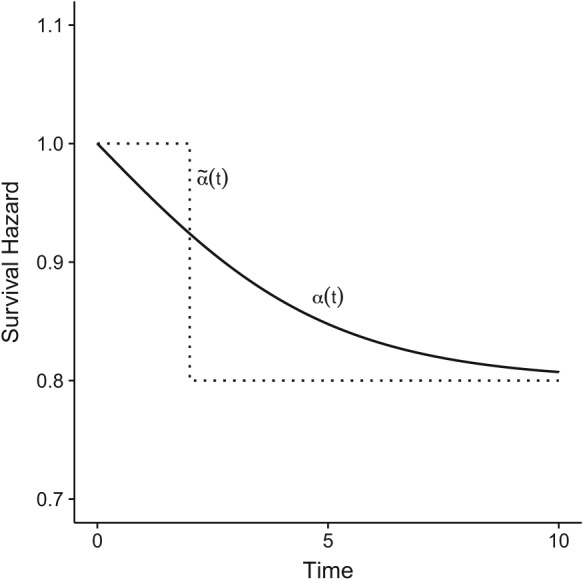
Comparison of the survival hazards $\tilde{\alpha }(t)$ for t_0_ = 2 specified a priori (dashed line) and the population survival hazard α(t) resulting from the illness‐death model without recovery of Figure 1 (solid line) in the presence of a time‐dependent covariate with, at most, one change over time. Details are given in Appendix B

In order to contrast simulation from a multistate perspective with the competing approaches when time‐dependent covariates are incorporated, we exemplary consider one time‐dependent exposure stochastic process *Y*(*t*) = **1**(*t* > *T*_0_) ∈ {0,1}. Here, *T*_0_ > 0 denotes the random time‐to‐exposure (TTE), and **1**(·) denotes the indicator function such that *Y*(*t*) becomes a left‐continuous dichotomous process with, at most, one switch over time (right panel in Figure 2). In event‐driven oncology trials, *T*_0_ commonly represents the time‐to‐progression. Furthermore, denote *T* as the time until death from any cause or, equivalently, as overall survival (OS). Exposure‐free survival (EFS) is given by the minimum of *T*_0_ and *T*. For simplicity, we assume that all individuals are not exposed at the time origin and completely observed, ie, we have no censoring or truncation. However, the arguments may be transferred to nondegenerated initial exposure distributions as well as covariates *Y*(*t*) with a finite number of categories, arbitrary switches, as well as to left‐truncated and/or right‐censored data. Note that, from a practical point of view, EFS ≤ OS and EFS = OS only if the patient dies without prior exposure. In order to account for this time‐dynamic pattern of events, one frequent approach is to use a Cox proportional hazards structure for OS incorporating exposure status as a time‐dependent covariate. For that purpose, let *α*_0_(*t*) be the survival hazard under non‐exposure and $\alpha_{0}(t)\cdot \exp(\beta )$ be the survival hazard under exposure, respectively. Here, exp(β) denotes the hazard ratio representing the effect of being exposed versus non‐exposed. In Appendix A, we show that under these specifications, the *population* survival hazard incorporating *Y*(*t*) can be expressed as

(6) $$\alpha (t)=\frac{P(Y(t)=0,T\geq t)}{P(T\geq t)}\cdot \alpha_{0}(t)+\frac{P(Y(t)=1,T\geq t)}{P(T\geq t)}\cdot \alpha_{0}(t)\cdot \exp\left(\beta \right),$$

with *α*(*t*)≡0 if *P*(*T* ≥ *t*) = 0 (see solid line in Figure 3 for an example). Note that relation (6) is a nonrandom function in time. In other words, the survival hazard *α*(*t*) decomposes into a weighted mixture of *α*_0_(*t*) and $\alpha_{0}(t)\cdot \exp(\beta )$ with weights corresponding to the prevalence of being (not) exposed at *t*. We aim to develop a model complying with relation (6), taking advantage of the multistate framework of Section 2. Let *X*_*t*_ ∈ {0,1,2} be a time‐inhomogeneous Markov process fulfilling the relation

(7) $$X_{t-}=\left\{\begin{array}{cc} 0, & \text{if}\;Y(t)=0,\;T\geq t,\\ 1, & \text{if}\;Y(t)=1,\;T\geq t,\\ 2, & \text{if}\;t>T. \end{array}\right.$$

In other words, *Y* and *X* coincide while the individual is still alive apart from the random time *T*_0_, where *X*(*T*_0_) = 1, but *Y*(*T*_0_) = 0 and *Y*(*T*_0_ + ) = 1 to make *Y* left‐continuous (see Figure 2). The reason is that multistate processes require right‐continuous sample paths.^33^ The underlying death hazards are defined as *α*_02_(*t*) = *α*_0_(*t*) and $\alpha_{12}(t)=\alpha_{0}(t)\cdot \exp(\beta )$ such that the Cox proportional hazards structure is satisfied. If we additionally allow for an “exposure hazard” *α*_01_(*t*), we end up with an illness‐death model without recovery, as illustrated in Figure 1. This formulation leads to a model with one initial state, one intermediate state (“exposure”), and one absorbing state (“overall death”). Here, exposure is modeled as a “competing risk” for death without prior exposure, and we allow for death after being previously exposed. We note that the waiting time in the initial state is EFS, ie, $\text{EFS}=\min\{t\in R_{+}:X_{t}\neq 0\}$, and the waiting time until the absorbing state is OS, ie, $T=\min\{t\in R_{+}:X_{t}=2\}$. All in all, relation (7) incorporates the values of the dichotomous time‐dependent covariate *Y*(*t*) as separate transient states and captures the random exposure behavior through *α*_01_(*t*). This intuitively describes the time‐dynamic development of *Y* and *T* over the course of time and highlights the connection between multistate models and time‐dependent exposures.^20^

In Section 4, we explain how such more complex multistate data can be generated following fundamental arguments of Gill and Johansen.^34^ The appeal is that the hazard specifications and the underlying data‐generating algorithm lead to quantities having an interpretation on the population level. More precisely, the proposed approach complies with (6) and implies that the expected proportion of individuals becoming exposed up to time *t* is given by the cumulative incidence function

(8) $$P(\text{EFS}\leq t,X_{\text{EFS}}=1)=\overset{t}{\underset{0}{\int }}\exp\left(-\left(A_{01}(u)+A_{02}(u)\right)\right)\cdot \alpha_{01}(u)du.$$

In contrast, one popular simulation technique in this setting independently generates TTE as well as dummy survival times (called OS_1_ and OS_2_) from common parametric distributions (eg, exponential^48^ or Weibull^49^). Then, one defines EFS as the minimum of OS_1_ and TTE. Finally,

(9) $$\text{OS}=\left\{\begin{array}{cc} \text{EFS}, & \text{if EFS}\neq {\text{TTE, ie, if OS}}_{1}\leq \text{TTE},\\ {\text{EFS+OS}}_{2}, & \text{if EFS}=\text{TTE, ie, if TTE}<{\text{OS}}_{1}. \end{array}\right.$$

Obviously, this procedure is based on a sampling space impossible in real life with unclear interpretation: first, a patient may die twice (at OS_1_ and at TTE + OS_2_ if TTE<OS_1_). Second, *each* individual is supposed to be exposed at *some time* TTE, which may not be in line with the population quantity in relation (8). For instance, we believe that it is somehow unrealistic (and not desired from a patient's perspective) to assume, for instance, a probability of progression in 100% of the study cohort. Third, death may preclude (“censor”) the observation of the individual exposure time, although it is unclear how to interpret, eg, time‐to‐latent‐progression for a patient that has died. Another drawback is that a latent structure does not discourage improper statistical analyses (cf Supporting Section S1). One prominent example is false Kaplan‐Meier–type analyses in competing‐risk settings.^61^ Contrary to this, the proposed approach outlined in Section 4 exclusively generates the real‐world times EFS and OS, but neither latent TTE after OS if OS = EFS nor latent OS_1_ if TTE<OS_1_. In‐depth discussions regarding the plausibility and identifiability of latent times can be found elsewhere.^15^, ^16^, ^17^, ^18^, ^33^ Nevertheless, a comparison of both data‐generating procedures shows that they lead (on average) to the same and correct OS specification (cf Supporting Section S1). For a mathematical proof, see elsewhere.^62^

Related suggestions^4^, ^6^, ^7^, ^8^, ^9^, ^10^, ^11^, ^12^, ^13^ formalize the survival hazard as

(10) $$\tilde{\alpha }(t|\bar{Y}(t))=1(t\leq T_{0})\cdot \alpha_{0}(t)+1(t>T_{0})\cdot \alpha_{0}(t)\cdot \exp(\beta ),$$

where $\bar{Y}(t))=\{Y(u);0\leq u\leq t\}$ is the exposure history up to time *t*.

As above, the algorithms first draw TTE (for instance, *t*_0_) such that (10) is interpreted *individually* for a given *T*_0_ = *t*_0_. A specific realization is given as a dashed line in Figure 3. Subsequently, the survival time *T* is generated from

(11) $$P(T>t|T_{0}=t_{0})=\left\{\begin{array}{cc} \exp\left(-\overset{t_{0}}{\underset{0}{\int }}\alpha_{0}(u)du\right), & \text{if}\;t\leq t_{0},\\ \exp\left(-\left(\overset{t_{0}}{\underset{0}{\int }}\alpha_{0}(u)du+\overset{t}{\underset{t_{0}}{\int }}\alpha_{0}(u)\cdot \exp(\beta )du\right)\right), & \text{if}\;t>t_{0}, \end{array}\right.$$

by, for example, applying its inverse to random draws of a standard uniform distribution. Due to the same reasons as above, we end up in a sampling space impossible in real life.

Another complication is that a translation of the survival hazard (10) into survival probabilities (11) is only reasonable for “external” time‐dependent exposures such as environmental factors, because they satisfy the formal relationship^14^

(12) $$P\left(u\leq T<u+du|T\geq u,\bar{Y}(u)\right)=P\left(u\leq T<u+du|T\geq u,\bar{Y}(t)\right)$$

for all *u*,*t* such that 0 < *u* ≤ *t*. In particular, the survival hazard at time *u* is intimately linked to the observed covariate history up to time *u*, but the occurrence of a failure in [*u*,*u* + *du*) does *not* depend on the future exposure status at a later time *t*. The reason is that, conditioning on the a priori generated exposure time *t*_0_ (or, equivalently, on the entire exposure trajectory $\bar{Y}(\infty )$), the conditional survival distribution (11) becomes $P(T>t|\bar{Y}(\infty ))$, which is equal to $\exp(\int_{0}^{t}\tilde{\alpha }(u|\bar{Y}(u))du)$ under model (12). The latter has the usual survival function interpretation for a given external covariate path. Note that, in this case, (6) is the (nonrandom) expectation with respect to the distribution of *T*_0_ of the random quantity (10) (cf Appendix A), which is one way to see why this procedure and our proposal lead to the same and correct data structure, but interpretations of the data‐generating mechanisms (particularly of the exposure hazards) differ.

In contrast, condition (12) does *not* hold for “internal” time‐dependent exposures such as progression in oncology. The reason is that their trajectories carry direct information about the failure time. More formally, $P(T\geq t|\bar{Y}(t))=1,$ *provided* that *Y*(*t* − ) exists, which means that it “requires survival of the individual for its existence” (see the book of Kalbfleisch and Prentice^14^). From a simulation point of view, conditioning on $\bar{Y}(\infty )$ and subsequently generating *T* from (11) would violate the fundamental principle to not condition on future internal exposure status.^63^, ^64^ The problem is not the Cox model structure of relation (10) but that its transformation via (11) lacks a meaningful survival function interpretation. Appendix C shows that, in this simplified survival situation described by a Cox proportional hazard structure, simulation via a multistate perspective and via relation (11) nevertheless (on average) leads to the same and correct OS specification even in the presence of an internal time‐dependent covariate. However, the benefits of our proposal are self‐evident: the time‐dependent exposure process *Y*(*t*) is not generated a priori but part of the data‐generating mechanism via *α*_01_(*t*). This makes simulation more “natural” compared to the abovementioned competing approaches, because only the real‐world times (in the present example: EFS and OS) are generated, hypothetical latent times are avoided, and the natural order of the events is guaranteed. Thus, its parsimony follows the principles of Occam's razor,^42^ whereas still allowing for flexible parameterizations as in, eg, the work of Crowther and Lambert.^35^ Furthermore, the survival hazard (6) as well as the cumulative incidence function (8) are population average quantities with a clear interpretation. These are the reasons why we believe that simulating data in line with fundamental principles of the analyses—such as not conditioning on the future—is desirable and should be preferred.

## 4 EMPIRICAL SIMULATION OF COMPLEX MULTISTATE DATA BASED ON REAL DATA EXAMPLES

In order to simulate complex time‐to‐event data as in relation (7), we take up the hazard‐based simulation algorithm mathematically established by Gill and Johansen.^34^ The fundamental probabilistic result is that only the transition hazards are required to completely regulate the stochastic behavior of the multistate process. Following the work of Beyersmann et al,^33^ one multistate trajectory can be generated as follows.

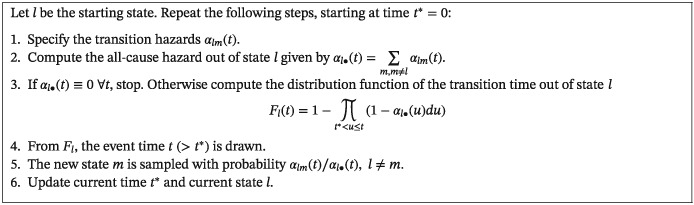

Note that, in the presence of continuous hazards, the product integral given in step 3 is equivalent to $\exp(-A_{l•}(t)+A_{l•}(t^{*}))$, where $A_{l•}(t)=\int_{0}^{t}\alpha_{l•}(u)du$ is the cumulative all‐cause hazard out of state *l*. Step 4 may be realized by means of the inversion method or more powerful procedures suggested elsewhere.^4^ The algorithmic perspective shows how the transition hazards (1) construct multistate processes as a successive nested series of competing risks experiments. It has to be emphasized that the algorithm does not condition on future events and avoids latent times. The reasons are, on the one hand, a stopping criterion when an absorbing state is reached and, on the other hand, the fact that the transition type is determined *after* the event time has been generated. Consequently, the generation of (internal) time‐dependent covariate processes is not done a priori but is part of the data‐generating mechanism. Independent right‐censoring and left‐truncation mechanisms can straightforwardly be incorporated.^33^ For instance, the algorithm can be adapted to administrative censoring (type I), event‐driven censoring (type II), and random censoring (ie, censoring times are stochastically independent of *X*_*t*_). State‐dependent censoring can be realized by adding “censoring states” in the multistate model with underlying state‐dependent censoring hazards. If one of these states is chosen in step 5, the individual is right‐censored. Similar arguments apply to left‐truncation.^65^

Step 1 of the algorithm typically assumes common parametric models, where we have analytical expressions for the transition hazards (cf the work of Klein and Moeschberger^66^ for an overview). Nontrivial parametric approaches are discussed in the work of Crowther and Lambert.^35^ However, simulation studies are often motivated by a specific data example; thus, parameterizations may be too restrictive or methodologically expensive. Instead, we outline an “empirical” version of the algorithm, which does not require closed forms for the hazards. The usage of nonparametric distributions provided by, for example, histograms has already been suggested by Sylvestre and Abrahamowicz in a passing comment.^19^ The key idea is to work with the estimated cumulative hazards, which may be based on *published* data.^45^, ^47^, ^67^ More precisely, our proposal only needs the increments of the Nelson‐Aalen estimators $\Delta {\hat{A}}_{lm}(t)={\hat{A}}_{lm}(t)-{\hat{A}}_{lm}(t-)$ introduced in (5) and used instead of *α*_*lm*_(*t*)*dt*. They can either be derived from the original individual patient data using standard software or even deduced from the respective cumulative hazards plots given in publications (see Section 5.2 for details). The distribution function of the transition time *F*_*l*_(*t*) in step 3 is estimated by the empirical Kaplan‐Meier–type distribution function ${\hat{F}}_{l}(t)=1-\prod_{t^{*}<u\leq t}(1-\Delta {\hat{A}}_{l•}(u))$, whose increments are subsequently used for a multinomial experiment to determine the event time out of state *l*. A practical complication is that ${\hat{F}}_{l}$ may spend less than 100% of probability mass in the presence of right‐censoring in the original study. In that case, one assigns the remaining probability mass to a timepoint beyond the largest observed time and always censors corresponding individuals, if this artificial time is drawn. Under the random censorship model, other censoring times can, for instance, be derived from the censoring Kaplan‐Meier estimator. An implementation of the algorithm is in the function mssample of the R package mstate.^52^, ^53^

Of course, this empirical formulation is flexible enough to account for modeling assumptions, eg, following the framework of Section 3, ${\tilde{A}}_{12}(t):={\hat{A}}_{02}(t)\cdot \exp(\beta )$. Notable is the fact that the simulated transition times follow a discrete time grid. Consequently, the distribution of the transition times becomes more and more discrete if the number of simulated patients distinctly exceeds the original sample size.

## 5 APPLICATION

### 5.1 Study example

The following simulation studies utilize the published CSL 1 trial presented in example 1.3.12 in the work of Andersen et al.^50^ It considers 488 liver cirrhosis patients from a randomized clinical trial comparing the hormone “Prednisone” (*n* = 251) with an inactive placebo treatment (*n* = 237). The study aim was to assess prolonged survival in Prednisone‐treated patients. The timescale of interest was “time since randomization” in days. Individuals were followed until death, with subjects being censored at the end of the trial. We focus on the effect of the “prothrombin index,” which is an indicator for liver functionality. The present study dichotomizes the index into “abnormal” (state 1) and “normal” (state 0). The main practical issues are that the prothrombin status is an internal time‐dependent covariate and that patients can switch between normal and abnormal prothrombin levels for arbitrary times during follow‐up. This is captured by an illness‐death multistate model *with* recovery, which jointly incorporates the time‐dependent abnormal prothrombin exposure and the survival outcome “death” (absorbing state 2). A graphical illustration would be as in Figure 1, but with an additional arrow from state 1 to state 0 and corresponding hazard *α*_10_(*t*) to account for possible multiple prothrombin status switches over time (see figure I.3.6 in the work of Andersen et al^50^).

We focus on Prednisone‐treated patients. Here, 108 patients initially entered the study with normal prothrombin levels and 143 patients with abnormal ones. Moreover, 92 patients died under exposure and 50 patients under no exposure, respectively. There were 109 censored observations. In total, 290 switches between the two prothrombin levels were observed (cf table I.3.7. in the work of Andersen et al^50^).

### 5.2 Proof of concept

A first simulation study is performed in order to investigate whether the empirical simulation technique approximately recovers the original quantities, where the study‐based Nelson‐Aalen estimators act as the “true” benchmarks. In this specific case, individual patient data are given by the prothr data set included in the R package mstate 52, 53 such that the (increments of the) Nelson‐Aalen estimators can directly be reconstructed (see left panel in Supporting Figure S2). If these data had not been available, they could have been derived from figures IV.4.10 and IV.4.11 published in the work of Andersen et al^50^ either manually or using developments in image processing software recently applied in the context of survival analysis.^67^, ^68^ The initial empirical proportions of patients at the time origin are *π*_0_ = 0.43 (normal) and *π*_1_ = 0.57 (abnormal).^69^ Although the algorithm may lead to censored observations in this example (since the original data include censored observations such that ${\hat{F}}_{l}$ may spend less than 100% of probability mass), we additionally superimpose random right‐censoring times. They are generated according to the increments of the study‐based censoring Kaplan‐Meier estimator (right panel in Supporting Figure S2). Censoring Kaplan‐Meier estimators are typically not published, but the number of patients at risk may be used to obtain the desired information. We simulate 2000 data sets, each including 251 patients as in the original study. For the proof of concept, we compare the estimated state occupation probabilities obtained from the simulated data sets with the respective study‐based quantities. The reason for considering state occupation probabilities is 2‐fold: on the one hand, they are relevant outcomes in real practical analyses; on the other hand, they are complex functionals incorporating the initial state distributions and the Aalen‐Johansen estimator $\hat{P}(0,t)$ introduced in (4). For instance, the “true” (study‐based) estimated probability to be alive with normal prothrombin index is

(13) $$\hat{P}(\text{alive and normal PI at}\;t)=\pi_{1}\cdot {\hat{P}}_{10}(0,t)+\pi_{0}\cdot {\hat{P}}_{00}(0,t),$$

where ${\hat{P}}_{lm}$ is the (*l*,*m*)th entry of $\hat{P}$. Note that if the increments of the Nelson‐Aalen estimators are known, the state occupation probabilities are directly obtained via relation (4).

**Figure 4:**
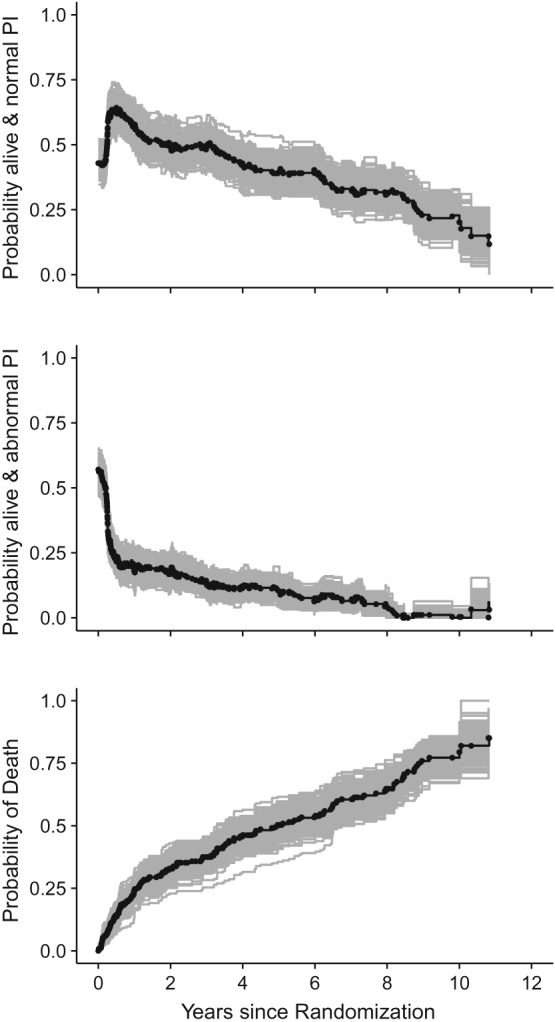
Simulation algorithm—proof of concept. Solid black lines are the “true” (study‐based) state occupation probabilities, and solid gray lines are 300 randomly selected state occupation probabilities from the simulation. The averages of the simulated state occupation probabilities are drawn as black dots. PI, prothrombin index

In Figure 4, we separately plotted the three “true” probabilities from the study example (solid black lines) together with the respective averages over the estimated probabilities of the simulated data sets (black dots). For illustrative purposes, 300 randomly selected simulated curves are included (gray lines). In all three subfigures, the averages over the simulated quantities are hardly distinguishable from the true underlying quantities even for the latest timepoints. Consequently, the proposed simulation technique, on average, generates data consistent with the original quantities. We also observe that the simulated curves only have jumps at the original transition times, but not in between. This characteristic is triggered by the empirical nature of the algorithm.

A second investigation addresses the coverage probabilities of confidence intervals for varying sample sizes. For that purpose, we use the same specifications as in the previous simulation study, but additionally consider the sample sizes 50, 100, 200, 500, 1000. For each scenario, we again simulate 2000 data sets. For each transition, we check whether the 95% log‐log transformed confidence interval for the transition probabilities derived from the simulated data set covers the true study‐based quantity ${\hat{P}}_{lm}(0,t)$. The study‐based estimated transition probabilities are visualized in Supplementary Figure S3. Variance estimation is based on the Aalen‐type estimator.^50^ Table 1 presents the resulting coverage probabilities computed at the exemplary timepoints *t* = 378, 1800, 2700, and 3200 days. These times are chosen according to the 25%, 50%, 65%, and 75% quantiles of the OS time distribution provided in the lowest panel of Figure 4.

**Table 1:** Simulation results in terms of coverage probabilities for the proof of concept in Section 5.2. Coverages are computed for each (study‐based) transition probability ${\hat{P}}_{lm}(0,t)$ evaluated at different timepoints and sample sizes

		Coverage Probability, %			
*n*	*t*	${\hat{P}}_{01}(0,t)$	${\hat{P}}_{02}(0,t)$	${\hat{P}}_{10}(0,t)$	${\hat{P}}_{12}(0,t)$
50	378	94.9	92.5	93.8	94.0
	1800	92.6	92.6	93.6	92.8
	2700	71.5	90.7	92.7	91.5
	3200	14.8	90.0	92.0	90.5
100	378	94.6	94.8	95.1	94.3
	1800	93.3	94.2	93.7	93.0
	2700	87.6	94.2	94.3	93.8
	3200	28.5	93.0	94.0	93.1
200	378	95.2	95.0	94.5	94.9
	1800	94.6	93.8	94.8	93.9
	2700	92.4	94.1	94.0	94.0
	3200	50.6	94.5	94.1	94.4
251	378	95.3	95.2	94.7	95.0
	1800	94.0	94.4	94.6	94.2
	2700	92.5	94.4	94.3	94.6
	3200	59.0	93.3	94.9	93.4
500	378	96.0	93.9	94.8	93.5
	1800	95.1	94.9	94.7	94.4
	2700	94.0	94.4	94.4	94.2
	3200	82.4	94.5	94.5	94.1
1000	378	95.2	94.6	94.5	94.7
	1800	95.5	94.9	94.5	93.9
	2700	94.2	94.2	95.2	95.2
	3200	94.1	94.0	94.7	94.6

We observe that coverage probabilities for the original sample size of 251 patients nicely approach the confidence level of 95%, except for the study‐based quantity ${\hat{P}}_{01}$ evaluated at the latest timepoint. This is due to the fact that it is almost zero (see Supplementary Figure S3). For the smaller sample sizes of 100 and 200 individuals, the confidence level is also well approximated, but a further reduction yields too liberal statements. Again, results for later timepoints should not be over‐interpreted. In order to obtain approximately 95% coverage even for the latest timepoint, the sample size has to be increased up to 1000 individuals.

### 5.3 Performance of bootstrapped confidence intervals for state occupation probabilities

In the previous subsection, functionals of the study‐based Nelson‐Aalen estimators acted as the true benchmarks. These are generally step functions on a time‐discrete grid. The present section now utilizes the proposed simulation algorithm to build bootstrap data sets in order to assess the uncertainty for the estimated state occupation probabilities, but now with regard to parametrically specified time‐continuous hazards. For that purpose, we assume an illness‐death model with recovery as in the previous section with (true) constant hazards *α*_01_(*t*)≡0.0005, *α*_02_(*t*)≡0.0002, *α*_10_(*t*)≡0.002, and *α*_12_(*t*)≡0.0012. The quantities correspond to the transition‐specific incidence rates corresponding to the CSL 1 trial. The initial states are determined by a binomial experiment with probabilities 0.43 (state 0) and 0.57 (state 1) in line with the parameter values of Section 5.2. Censoring times are uniformly distributed between 0 and 4400.

We consider the sample sizes 50, 100, 200, 251, and 500 patients and timepoints *t* = 378, 500, 1000, 1800, 2700, 3200. For each sample size, we simulate 1000 studies employing the non‐empirical simulation algorithm described in Steps 1‐6 of Section 4. Within each study, the resulting Nelson‐Aalen estimators are used to generate 1000 bootstrap data sets by means of the proposed empirical simulation procedure. The 95% bootstrap confidence interval margins for the state occupation probabilities *P*(*X*_*t*_ = *j*) are set to the corresponding 2.5% and 97.5% quantiles of the corresponding 1000 bootstrapped state occupation probabilities.

Simulation results in terms of coverage probabilities are summarized in Table 2. We observe that bootstrap confidence intervals show satisfactory performances for *P*(*X*_*t*_ = 1) and *P*(*X*_*t*_ = 2) and early timepoints *t*, even for small numbers of simulated individuals. Corresponding intervals for *P*(*X*_*t*_ = 0) tend to be slightly too conservative. Moreover, gradually increasing the number of simulated patients lead to suitable coverages close to the confidence level of 95% even for later timepoints. We also note that coverage probabilities are consistently smaller than 95% for the two latest timepoints throughout all sample sizes. This is due to almost negligible true state occupation probabilities and/or the probabilities to be at risk as a result of the simulation configuration (cf Supplementary Table S1). This generally makes prediction difficult.

**Table 2:** Coverage probabilities for the simulation study in Section 5.3

		Coverage Probability, %		
*n*	*t*	*P*(*X*_*t*_ = 0)	*P*(*X*_*t*_ = 1)	*P*(*X*_*t*_ = 2)
50	378	97.4	95.1	94.0
	500	97.4	94.5	94.9
	1000	95.5	93.1	94.8
	1800	92.9	88.8	92.3
	2700	90.3	66.8	90.9
	3200	89.1	68.7	91.2
100	378	97.2	94.7	94.2
	500	96.5	94.3	94.9
	1000	94.1	94.3	94.6
	1800	93.6	92.5	94.1
	2700	92.1	87.2	92.8
	3200	90.3	74.8	91.9
200	378	96.8	95.4	94.9
	500	95.7	95.0	95.0
	1000	96.1	95.2	95.4
	1800	93.8	91.9	94.2
	2700	92.6	89.5	92.9
	3200	94.1	89.1	93.7
251	378	97.3	94.9	94.4
	500	95.4	94.5	95.9
	1000	96.7	95.0	96.4
	1800	94.9	92.1	95.0
	2700	92.8	89.8	94.0
	3200	92.3	87.6	93.2
500	378	96.8	95.3	96.4
	500	96.7	95.0	95.6
	1000	94.9	95.7	95.0
	1800	94.9	93.9	95.8
	2700	92.1	91.4	93.3
	3200	91.8	89.4	91.6

Overall, we can conclude that the proposed empirical simulation algorithm may be used to assess uncertainty by means of model‐based bootstrap data sets as originally suggested.^47^ The novelty is that the technique does work without individual patient data, which is not the case, for example, Efron's nonparametric bootstrap requiring all individual multistate trajectories.

## 6 DISCUSSION

We have proposed an empirical resampling technique for complex time‐to‐event data based on an empirical multivariate hazard measure. Our multistate approach follows a probabilistic construction suggested in the work of Gill and Johansen,^34^ which is the intuitive way of how to describe the time‐dynamic pattern of (internal qualitative) time‐dependent covariates and events over time by avoiding hypothetical latent times and sampling spaces impossible in real life. This parsimonious perspective not only complies with Occam's razor but also provides for a model specification of time‐dependent exposures and survival that has a proper interpretation as a population‐level summary. Furthermore, the algorithm can serve as an operational tool in order to explain why multistate frameworks are helpful in real data analyses. Overall, we argued that simulation algorithms should be plausible (and not just the data structure) and in line with fundamental principles of the time‐to‐event methodology—such as not conditioning on the future following the same assumptions as the statistical analysis.^64^ These arguments are important if simulations are used for sample size calculations, eg, by adapting recent context‐related proposals,^44^, ^70^ and would be helpful in describing the required data‐generating model in a trial protocol.

Our application was restricted to an illness‐death model with (without) recovery, jointly modeling survival in the presence of one time‐dependent dichotomous covariate. Adapting the state space, the flexibility of the multistate framework allows for complex event histories, including more than one (qualitative) covariate, additional intermediate events, and competing endpoints. Continuous covariates may be incorporated either by decomposing the values into a finite number of categories or by sufficiently inflating the state space of the model. However, the number of states has to be a trade‐off between data availability and clinical expertise in order to guarantee a sufficiently large number of events. A topic for future research is to simulate via “joint models” for longitudinal responses and time‐to‐event data.^71^ Another very recent work discussing the connection between time‐dependent covariates and multistate models is that by Le‐Rademacher et al.^25^ These authors also use simulations but only report results for time‐constant hazards. In addition, details on the simulation algorithm are not given.

This paper proposed an empirical analogue of the Gill and Johansen algorithm. One attractive feature is that it may be based on published material. The quantities that need to be extracted from the publications of medical studies are (i) the (increments) of the estimated cumulative hazards, (ii) the initial distributions, and, ideally, (iii) information on left‐truncation and/or censoring mechanisms (eg, in terms of the censoring Kaplan‐Meier estimator or the risk sets). In standard survival settings, the Nelson‐Aalen estimator can be derived from the Kaplan‐Meier estimator typically presented in publications. The reason is the one‐to‐one relation between hazards and probabilities.^33^ A prominent example is randomized clinical trials in oncology with a composite as a primary endpoint. Studies of more complex time‐to‐event settings are just starting to report the transition‐specific Nelson‐Aalen estimators (see elsewhere for examples^23^, ^27^, ^31^, ^32^, ^72^, ^73^). The major challenge for the empirical algorithm is to construe the increments from the published figures. One possibility is to do it manually. A topic for future research is the application of image processing software, which has recently been applied in the context of survival analysis.^67^, ^68^

Our empirical procedure enables simulation studies plausibly mimicking complex real‐world time‐to‐event data (see elsewhere for recent applications^74^, ^75^). This may also be relevant for sample size calculations, when historical data (without access to individual patient data) would be incorporated. Furthermore, it is a simple technique to make trial data publicly accessible without losing its overall properties. This is a concern whenever copyright restrictions do not allow the use or distribution of the original data. In principle, all competing simulation approaches mentioned in the Introduction may also be applicable without individual patient data; however, the parameters should be specified such that the motivating study is well approximated. One solution is the application of preprocessing procedures such as parametric assumptions or smoothing techniques (applied to each single transition hazard). The disadvantage is that these may be too restrictive and/or computationally expensive.

We found that our proposal approximates the empirical hazard measures well and can be utilized to build bootstrap data sets in order to assess uncertainty in parameter estimation even without access to individual patient data. This is contrary to standard resampling techniques such as Efron's nonparametric bootstrap. The time‐discrete perspective used within the algorithmic framework is a typical situation in longitudinal studies and makes it comparable to competing approaches in terms of computational costs; however, the distribution of the event times becomes more and more discrete if the number of simulated patients distinctly exceeds the original sample size. Although it seemed not to be an issue in the present simulation studies, further investigations are needed to assess a deviation of the time‐continuity assumption.

The framework of Gill and Johansen makes a time‐inhomogeneous Markov assumption, but the flexibility of the algorithm allows for non‐Markov settings (eg, by including frailties or past information such as state arrival times in Cox models for the transition hazards) as well as nonproportional settings (eg, Aalen's additive model^33^). Our proposal has been implemented by one of us (HP) in the R package mstate, and the code has been independently validated by a second coauthor (TB), so that applications in more general models are readily available for users of R. Example code is provided as web‐based Supporting Information.

## Supporting information




**SupportingInformation.pdf** includes the comparison of two simulation algorithms as well as Supplementary Figures S1 and S2 and Supplementary Table S1.

SIM_8177‐Supp‐0001‐SupportingInformation.pdf

SIM_8177‐Supp‐0002‐ExampleCode.R

## APPENDIX A

### A.1 SURVIVAL HAZARD IN THE PRESENCE OF AN INTERNAL TIME‐DEPENDENT EXPOSURE

As in Section 3, let *T* be the time‐to‐death, and assume an internal left‐continuous exposure process *Y*(*t*) ∈ {0,1} with, at most, one jump at the random timepoint *T*_0_ > 0. Then, the law of total probability implies

$$\begin{array}{ll} \alpha (t)dt & =P\left(T\in dt|T\geq t\right)=\frac{P\left(T\in dt,Y(t)=0\right)+P\left(T\in dt,Y(t)=1\right)}{P\left(T\geq t\right)}\\ & =\frac{P\left(T\in dt|Y(t)=0,T\geq t\right)\cdot P\left(Y(t)=0,T\geq t\right)+P\left(T\in dt|Y(t)=1,T\geq t\right)\cdot P\left(Y(t)=1,T\geq t\right)}{P\left(T\geq t\right)}\\ & =\frac{P\left(Y(t)=0,T\geq t\right)}{P\left(T\geq t\right)}\cdot \underset{=:\alpha_{02}(t)dt}{\underbrace{P\left(T\in dt|Y(t)=0,T\geq t\right)}}+\frac{P\left(Y(t)=0,T\geq t\right)}{P\left(T\geq t\right)}\cdot \underset{=:\alpha_{12}(t)dt}{\underbrace{P\left(T\in dt|Y(t)=1,T\geq t\right)}}. \end{array}$$

Note that if *Y*(*t*) is external, *α*(*t*) is equal to the expectation of the right‐hand side of (10) for *α*_02_(*t*) = *α*_0_(*t*) and $\alpha_{12}(t)=\alpha_{0}(t)\cdot \exp(\beta )$, because, for instance,

$$\frac{P\left(Y(t)=0,T\geq t\right)}{P\left(T\geq t\right)}=P\left(Y(t)=0|T\geq t\right)=P\left(Y(t)=0\right)=E\left(1\left(t\leq T_{0}\right)\right).$$

If we now introduce a stochastic process *X*_*t*_, which is not necessarily Markov, but fulfilling relation (7), we can distinguish the following cases.

1. If *X*(*t*) is time‐inhomogeneous Markov, then *α*_02_(*t*) and *α*_12_(*t*) are the usual transition hazards defined in (1).
2. If *X*(*t*) follows an illness‐death model without recovery as illustrated in Figure 1, then *α*_02_(*t*) is the usual transition hazard, but *α*_12_(*t*) only if the process is Markov. In particular, if *α*_02_(*t*) = *α*_0_(*t*) and $\alpha_{12}(t)=\alpha_{0}(t)\cdot \exp(\beta )$, we end up with relation (6).
3. In general, *α*_*l*2_,*l* ∈ {0,1} are *partly conditional* transition rates^76^ in that, eg, *P*(*T* ∈ *dt*|*Y*(*t*) = 1,*T* ≥ *t*) only conditions on the present covariate value but not on the entire covariate history of the process *Y*(*t*).

## APPENDIX B

### B.1 ILLUSTRATIVE EXAMPLE OF A DIFFICULT‐TO‐INTERPRET SURVIVAL HAZARD IN THE PRESENCE OF AN INTERNAL TIME‐DEPENDENT EXPOSURE

Following Figure 1, suppose an illness‐death model without recovery with underlying multistate process *X*_*t*_ fulfilling relation (7) and internal covariate process *Y*(*t*) ∈ {0,1}. Furthermore, assume that $P\left(Y(t)=0\right)=1$, ie, all individuals have the covariate value 0 at time *t* = 0. Let *α*_02_(*t*) = *α*_0_(*t*)≡1, $\alpha_{12}(t)=\alpha_{0}(t)\cdot \exp(\beta )$ with exp(β)=0.8 and *α*_01_(*t*)≡0.2. Then, the waiting time distributions in states 0 and 1 can be expressed as^51^

$$\begin{array}{ll} P_{00}(s,t) & =\exp\left(-1.2\cdot [t-s]\right)\;\text{and}\\ P_{11}(s,t) & =\exp\left(-0.8\cdot [t-s]\right). \end{array}$$

This implies that the transition probability from state 0 to state 1 can be written as

$$\begin{array}{ll} P_{01}(0,t) & =\overset{t}{\underset{0}{\int }}P_{00}(0,u)\cdot \alpha_{01}\cdot P_{11}(u,t)du\\ & =-0.5\cdot \exp\left(-0.8\cdot t\right)\cdot \left[\exp\left(-0.4\cdot t\right)-1\right]. \end{array}$$

Then, relation (6) simplifies to

$$\begin{array}{ll} \alpha (t) & =\frac{P(Y(t)=0,T\geq t)}{P(T\geq t)}+0.8\cdot \frac{P(Y(t)=1,T\geq t)}{P(T\geq t)}\\ & =\frac{P_{00}(0,t-)}{P_{00}(0,t-)+P_{01}(0,t-)}+0.8\cdot \frac{P_{01}(0,t-)}{P_{00}(0,t-)+P_{01}(0,t-)}, \end{array}$$

which is given as a solid black line in Figure 3. For comparison, the Figure also includes the corresponding survival hazard (10) for an exposure realization *t*_0_ = 2 (dashed black line).

## APPENDIX C

### C.1 FIRST SIMULATING THE TIME‐DEPENDENT COVARIATE TRAJECTORY LEADS TO THE CORRECT DATA STRUCTURE FOR TIME‐DEPENDENT EXPOSURES

Let *T*_0_ > 0 be the random time‐to‐exposure with an absolutely continuous distribution function $F_{T_{0}}(t)$ and density function $F_{T_{0}}(t)$. Then, the hazard rate of *T*_0_ is given by^51^

(C1) $$\alpha_{01}(t):=\frac{f_{T_{0}}(t)}{1-F_{T_{0}}(t)}.$$

As in Section 3, let *T* be the time‐to‐death with the underlying survival hazard (10). Using standard calculations, the probability to be alive and exposed corresponding to an a priori generation of the exposure time can be expressed as

$$\begin{array}{ll} P(t<T,t<T_{0}) & =\overset{\infty }{\underset{0}{\int }}P(t<T,t<s|T_{0}=s)dP^{T_{0}}(s)\\ & =\overset{\infty }{\underset{t}{\int }}P(T>t|T_{0}=s)f_{T_{0}}(s)ds\\ & \overset{s>t}{=}\overset{\infty }{\underset{t}{\int }}\exp(-A_{02}(t))f_{T_{0}}(s)ds\\ & =\exp(-A_{02}(t))\cdot (1-F_{T_{0}}(t))\\ & \overset{\left(C1\right)}{=}\exp(-A_{02}(t)-A_{01}(t)), \end{array}$$

where $A_{01}(t)=\int_{0}^{t}\alpha_{01}(u)du$ as defined in (2). This result is equivalent to the usual state occupation probability *P*(*X*(*t*) = 0) derived from the illness‐death model with recovery of Figure 1 and model (7) with exposure hazard *α*_01_(*t*) (cf relation (3.71) in the book of Aalen and Gjessing^51^), because we have *P*(*X*_0_ = 0) = 1.

Similar arguments lead to

$$\begin{array}{ll} P(T_{0}<t<T) & =\overset{\infty }{\underset{0}{\int }}P(s<t<T|T_{0}=s)dP^{T_{0}}(s)\\ & =\overset{t}{\underset{0}{\int }}P(T>t|T_{0}=s)f_{T_{0}}(s)ds\\ & \overset{s<t}{=}\overset{t}{\underset{0}{\int }}\exp\left(-A_{02}(s)-\left(A_{12}(t)-A_{12}(s)\right)\right)\cdot f_{T_{0}}(s)ds\\ & \overset{\left(C1\right)}{=}\overset{t}{\underset{0}{\int }}\exp\left(-A_{01}(s)-A_{02}(s)\right)\cdot \alpha_{01}(s)\cdot \exp\left(-\left(A_{12}(t)-A_{12}(s)\right)\right)ds, \end{array}$$

which is equivalent to the state occupation probability being alive and exposed from the illness‐death multistate model (cf relation (3.73) in the book of Aalen et al^51^).

In summary, these results suggest that simulation using a multistate perspective and the competing simulation approach based on (11) lead to the same (and correct) data structure in terms of state occupation probabilities independent of the type of the incorporated time‐dependent covariate in our simplified setting.
