# Supplementary material for: Bootstrapping complex time‐to‐event data without individual patient data, with a view toward time‐dependent exposures
Source: Stat Med. 2019 Jun 4;38(20):3747–63. doi: 10.1002/sim.8177 (PMC6771611; doi:10.1002/sim.8177)
Supplement: Supplementary file 1 — SIM_8177‐Supp‐0001‐SupportingInformation.pdf [file SIM-38-3747-s001.pdf]

Received: Added at production | Revised: Added at production | Accepted: Added at production

DOI: xxx/xxxx

WEB-BASED SUPPORTING INFORMATION

Web-based Supporting Information: Bootstrapping complex time-to-event data without individual patient data, with a view towards time-dependent exposures

Tobias Bluhmki*[1] | Hein Putter[2] | Arthur Allignol[3] | Jan Beyersmann[1]

[1]Institute of Statistics, Ulm University, Helmholtzstrasse 20, 89081 Ulm, Germany
[2]Department of Medical Statistics and Bioinformatics, Leiden University Medical Center, P.O. Box 9604, 2300 RC, Leiden, The Netherlands
[3]Merck KGaA, Frankfurter Str. 250, 64293 Darmstadt , Germany

Correspondence
*Tobias Bluhmki, Institute of Statistics, Ulm University, Helmholtzstrasse 20, 89081 Ulm, Germany. Phone: +49 (0)731 50 33104. Fax: +49 (0)731 50 33110. Email: tobias.bluhmki@uni-ulm.de

Content:

Comparison of simulation algorithms
Figure S1–S3
Table S1

S1 | COMPARISON OF SIMULATION ALGORITHMS

In the following, we compare the algorithm of Fleischer et al. (2009)[1] outlined in Section 3 with the simulation algorithm based on a multistate perspective (cf. box in Section 4) established by Gill & Johansen[2]. As in Section 5 of Fleischer et al. (2009), we assume EFS $\sim$ Exp(0.284), $OS_1 \sim$ Exp(0.075), and $OS_2 \sim$ Exp(0.128). All patients start non-exposed. Following relation (9) we simulate 1000 studies each including 95 patients. For each study the Kaplan-Meier estimator for overall survival is computed. The results are given in the left panel of Figure S1. As proven in their Theorem 5, the algorithm on average generates data complying with the overall survival function given as red line. An exemplary excerpt of the data structure within each generated dataset is as follows:

```
id  TTE   OS1    OS2     EFS   OS
1   3.99  8.48   9.88    3.99  13.87
2   9.30  2.25   NA      2.25  2.25
3   3.73  8.05   13.06   3.73  16.79
4   5.06  86.12  1.74    5.06  6.81
5   6.99  24.54  10.56   6.99  17.55
6   1.61  10.48  3.68    1.61  5.28
```

We observe that the algorithm generates a latent structure assuming a time-to-exposure for *each* patient. Further, death may preclude exposure (e.g., for ID 2). Both lead to sampling spaces impossible in real life. Another drawback is that the structure does not discourage improper statistical analyses. For instance, a Kaplan-Meier analysis for the (hypothetical) times OS_1 or TTE may be performed. In other words, sampling spaces impossible in real life do not guide understanding and statistical modeling of such problems.

Using a multistate perspective based on an illness-death model without recovery, the above-mentioned specifications lead to a constant hazards setting, i.e., $\alpha_{01} = 0.284$, $\alpha_{02} = 0.075$, and $\alpha_{12} = 0.128$ with $P(X_0 = 0) = 1$. We now apply the algorithm of Gill & Johansen (1990) outlined at the beginning of Section 4 and also compute the Kaplan-Meier estimator for overall survival for each study. Following the right-hand panel of Figure S1, we see that both algorithms (on average) lead to the same and correct overall survival specification. The equivalence between Theorem 5 of Fleischer et al. (2009) and P_{02} using relation (3) has been proven elsewhere[3]. An exemplary excerpt of the data structure (as required for the `etm` package) within each generated dataset is as follows:

```
id entry  exit from to
```

```
1  0.00  3.24    0  1

1  3.24 15.60    1  2

2  0.00  3.40    0  1

2  3.40  9.38    1  2

3  0.00  1.67    0  2
```

It is obvious that this structure is more parsimonious following the principles of Occam's razor. In particular, the algorithm exclusively generates real world times. For instance, individuals 1 and 2 die after exposure, whereas individual 3 dies without prior exposure. This is in line with the population quantity in relation (8). In our opinion, the data structure also complies with the intuitive timing of events.

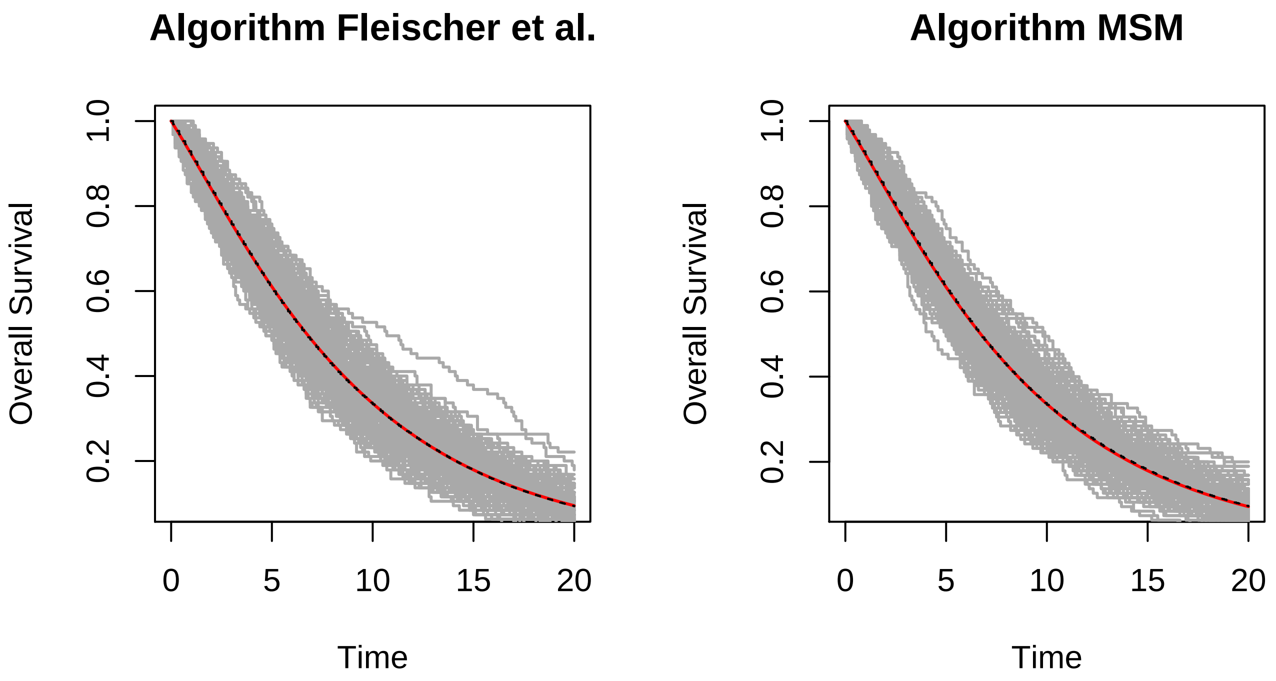

FIGURE S1 Comparison of the two algorithms (Left panel: Fleischer et al. (2009), right panel: Gill & Johansen (1990) based on an illness-death model without recovery). Red lines represent the true overall survival function, i.e., $1 - F_{\mathrm{OS}}$, where F_{OS} is given in Theorem 5 in Fleischer et. al (2009). The average of the 1000 Kaplan-Meier estimators are given as dashed black lines. The gray lines are 300 randomly selected Kaplan-Meier estimators from the simulations.

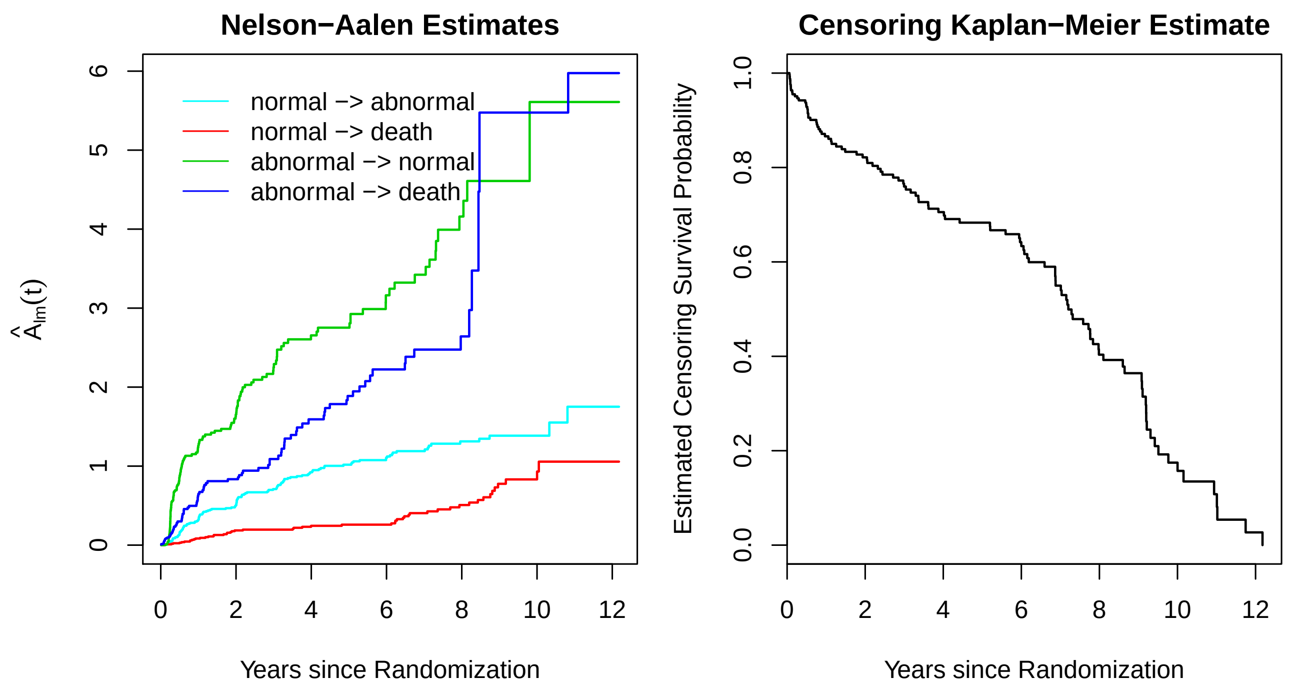

FIGURE S2 Nelson-Aalen estimators of the four cumulative transition-specific hazards in prednisone-treated patients of the study example in Section 5 (left panel); right panel shows the corresponding Kaplan-Meier estimator of censoring times.

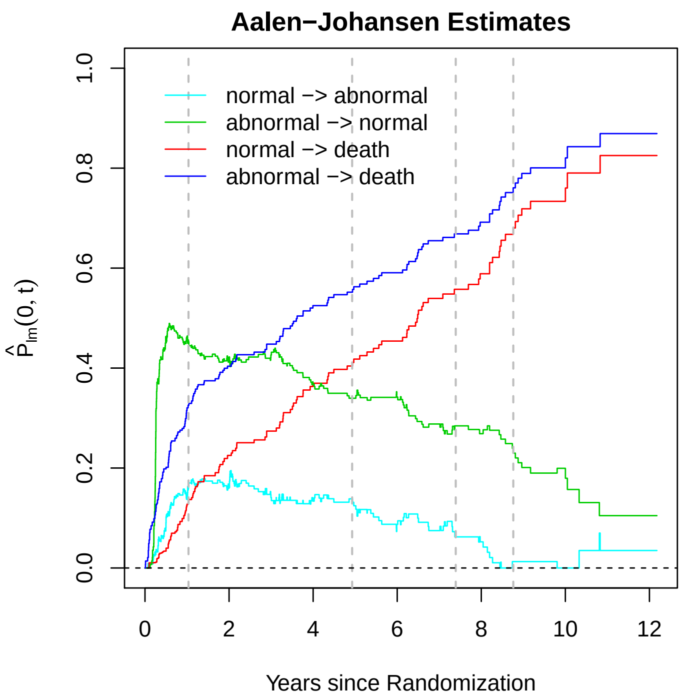

FIGURE S3 Aalen-Johansen estimators of the four transition probabilities in prednisone-treated patients of the study example in Section 5. Gray dashed lines indicate for which timepoints coverage probabilities are derived.

TABLE S1 True state occupation probabilities and probabilities to be at risk for the parameter specifications given in Section 6.2. The former is approximated via equation (3), which simplifies to a finite product for a very fine partition (increment 0.01 days) of the interval $[0, 4400]$ days. The latter is approximated by 1000 simulated datasets each including 1000 patients.

	State Occupation Probability			Probability at risk	
t	$P(X_t = 0)$	$P(X_t = 1)$	$P(X_t = 2)$	$P(Y(t) = 0)$	$P(Y(t) = 1)$
378	0.567	0.233	0.200	0.545	0.199
500	0.569	0.188	0.243	0.525	0.158
1000	0.514	0.108	0.378	0.409	0.083
1800	0.396	0.073	0.531	0.239	0.045
2700	0.290	0.053	0.657	0.115	0.021
3200	0.244	0.045	0.711	0.068	0.013

References

1. Fleischer F, Gaschler-Markefski B, Bluhmki E. A statistical model for the dependence between progression-free survival and overall survival. *Statistics in Medicine* 2009; 28(21): 2669-2686. doi: 10.1002/sim.3637

2. Gill RD, Johansen S. A survey of product-integration with a view toward application in survival analysis. *The Annals of Statistics* 1990; 18(4): 1501–1555.

3. Gaschler-Markefski B, Schiefele K, Hocke J, Fleischer F. Multi-state Models Used in Oncology Trials. In: Montfort vK, Oud J, Ghidey W., eds. *Developments in Statistical Evaluation of Clinical Trials*Berlin, Heidelberg: Springer Berlin Heidelberg. 2014 (pp. 283–304)
